# Transarterial embolization in inflammatory arthritis: will rheumatology shape the debate or inherit it?

**DOI:** 10.1093/rap/rkag125

**Published:** 2026-10-08

**Authors:** Francesco Ursini, Jacopo Ciaffi, Giancarlo Facchini, Piero Ruscitti, Roberto Caporali, Joel A Block

**Affiliations:** Medicine & Rheumatology Unit, IRCCS Istituto Ortopedico Rizzoli, Bologna, Italy; Department of Biomedical and Neuromotor Sciences, Alma Mater Studiorum University of Bologna, Bologna, Italy; Medicine & Rheumatology Unit, IRCCS Istituto Ortopedico Rizzoli, Bologna, Italy; Department of Biomedical and Neuromotor Sciences, Alma Mater Studiorum University of Bologna, Bologna, Italy; Diagnostic and Interventional Radiology Unit, IRCCS Istituto Ortopedico Rizzoli, Bologna, Italy; Department of Biotechnological and Applied Clinical Sciences, University of L’Aquila, L’Aquila, Italy; Department of Clinical Sciences and Community Health, University of Milan, Milan, Italy; Department of Rheumatology and Medical Sciences, ASST Pini-CTO, Milan, Italy; Division of Rheumatology, Department of Internal Medicine, Rush University Medical Center, Chicago, IL, USA

**Keywords:** embolization, arthritis, synovitis, monoarthritis, neovascularization, rheumatology

## Abstract

The expanding use of transarterial embolization for musculoskeletal pain, most commonly operationalized as genicular artery embolization in osteoarthritis, raises a broader question that rheumatology has not yet systematically addressed: might a vessel-targeted, image-guided intervention complement pharmacological care in selected patients with inflammatory arthritis? Despite advances in treat-to-target strategies, a single persistently inflamed joint can remain the dominant source of pain and disability while composite disease activity scores indicate target achievement. When that joint is refractory and systemic escalation is either disproportionate or infeasible, the therapeutic options are narrow and poorly evidenced. This review argues that the rheumatology community should be defining where, when and for whom transarterial embolization might have a legitimate place—its positioning within the inflammatory arthritis therapeutic landscape—rather than allowing this to be shaped, by default, through fragmented case-based experience and literature centred predominantly outside the discipline. Drawing on the neurovascular biology of synovitis, the osteoarthritis evidence and emerging reports in inflammatory conditions, we map the scenarios in which the question is starting to surface and outline what a deliberate positioning effort would require. In doing so, we recognise that no prospective, randomised or disease-specific data currently support transarterial embolization in this setting and that the available signal is limited to isolated case reports. However, defining a shared language now, before uncritical diffusion occurs, is a prerequisite for responsible investigation.

Key messages

- Transarterial embolization targets the hypervascularity sustaining synovitis, offering a vessel-directed approach to refractory monoarthritis.
- Evidence in inflammatory arthritis is confined to isolated case reports; no prospective data exist.
- Rheumatology should define this procedure’s place through cooperative protocols and registries before uncritical diffusion.

## The space that treatment frameworks leave empty

Treat-to-target has reframed inflammatory arthritis as a problem of disease activity measurement. What it has not resolved is what to do when the measure indicates that the target has been achieved but the patient remains substantially symptomatic—a familiar tension in routine rheumatology practice. Consider two scenarios, both illustrative of presentations encountered in clinical practice. The first is adapted from a case we have previously reported [1]; the second is a hypothetical vignette, representative of the checkpoint inhibitor–associated arthritis presentations managed across a range of rheumatology settings. Neither is offered as evidence of efficacy, but rather to ground the discussion in situations that recur across rheumatology clinics worldwide.

A 32-year-old man with long-standing juvenile idiopathic arthritis is well controlled on adalimumab. Following bilateral hip and ankle arthroplasties, his left ankle course was complicated by prosthetic infection requiring spacer implantation and revision surgery. The disease is now inactive at every site except the left ankle, where power Doppler ultrasound shows persistent synovitis. Intra-articular glucocorticoids provide brief relief. Systemic escalation risks destabilising otherwise adequate disease control and reintroducing infection risk in a previously infected prosthetic joint.

A 57-year-old woman receiving pembrolizumab for metastatic melanoma develops persistent monoarthritis of the right knee. Intra-articular injections and a course of oral glucocorticoids yield transient benefit. Methotrexate is discontinued for hepatotoxicity. Escalating to biologic or targeted synthetic DMARDs (bDMARDs and tsDMARDs) raises concerns about checkpoint inhibitor efficacy, and the entire inflammatory burden is confined to a single joint.

The clinical structure is the same in both scenarios: systemic disease controlled, one joint refractory, local options exhausted, systemic escalation disproportionate. Rheumatology’s therapeutic architecture was not built for this space. Composite disease activity scores can classify these patients as low disease activity or even remission while one joint continues to drive pain, limit function and concentrate structural risk [2]. The hazards of systemic escalation do not decrease with joint count; the incremental benefit often does [2]. Between repeated intra-articular glucocorticoid injections—an approach that often becomes progressively less effective—and intensification of systemic treatment or escalation to surgical synovectomy, there is no established intermediate strategy. This review asks whether transarterial embolization (TAE), an image-guided vessel-targeted intervention that has gained traction in OA, might one day find a carefully circumscribed place within this space. We are not arguing that it should. We are arguing that, if such a place exists, its positioning—for whom, at what point in the treatment pathway and in place of what—should be defined by the rheumatology community before practice shapes itself in its absence.

## Why the problem extends beyond refractory joints

Treat-to-target strategies have fundamentally changed the prognosis of inflammatory arthritis. Remission or low disease activity is now achievable for a substantial proportion of patients with RA, PsA and axial SpA [3–6]. Yet two gaps persist, and neither is adequately addressed by increasing systemic immunosuppression.

The first is the pain gap. Patient-reported pain in RA has remained essentially unchanged over 4 decades of therapeutic progress, despite dramatic reductions in objective synovitis [7]. This decoupling is not a measurement artefact. It reflects a biology of peripheral and central sensitisation that pharmacological immunosuppression addresses only partially and that calls for complementary strategies targeting the local joint environment [8, 9].

The second is feasibility. Some patients cannot escalate systemically, because of active infection or malignancy, major comorbidity or refusal of immunosuppression. Additionally, severe needle phobia, though underappreciated, is sufficiently prevalent to measurably shape care pathways in chronic disease populations [10, 11]. For these patients, the cumulative burden of an indefinite injection schedule can become the barrier itself. A single, image-guided procedure might be perceived as a different therapeutic proposition.

It is within this convergence—composite metrics that dilute joint-level burden, a persistent pain gap and barriers to conventional escalation—that vessel-targeted approaches are beginning to attract interest in inflammatory arthritis.

## From biological rationale to procedural reality

The rationale for TAE in musculoskeletal disease rests on a well-characterised phenomenon: pathological neovascularisation within the synovium, osteochondral channels and periarticular tissues sustains pain through aberrant nociceptive nerve sprouting and neurovascular coupling [12–14]. Power Doppler signal is not merely a marker of inflammation, it may represent a local vascular architecture that perpetuates synovitis even when systemic disease appears controlled [15, 16].

In inflammatory arthritis this substrate is, if anything, more pronounced than in OA. Inflammatory milieus rich in VEGF drive hyperaemia, vascular permeability and immune-cell trafficking in rheumatoid synovium [17]. Animal models show robust co-sprouting of calcitonin gene-related peptide–positive sensory fibres alongside new vascular channels—a structural correlate of pain amplification [18]. In humans, vascular and neural channels penetrate articular cartilage from subchondral bone through osteochondral conduits, with density correlating with symptomatic disease [19]. Rheumatoid synovium is neurochemically remodelled toward nociception, with substance P–dominant innervation patterns distinct from those in OA [20]. The synovium, in short, behaves as a remodelled neurovascular niche and the hypothesis for TAE is that selectively pruning its pathological microvascular supply attenuates both inflammatory trafficking and nociceptive sensitisation [21–23]. These preclinical models provide histological support for this hypothesis, albeit indirectly. In a rabbit model of surgically induced knee OA, genicular artery embolization reduced synovial proliferation, synovial hypertrophy and villous hyperplasia and significantly lowered synovial CD3^+^ T cell density and infiltrated area compared with sham, without altering structural cartilage damage [22]. In a rat adhesive capsulitis model, embolization reduced synovial microvessel and mononuclear inflammatory cell counts while improving physical activity, although chronic capsular fibrosis was unchanged [21]. A swine model of papain-induced synovitis has further confirmed the feasibility and short-term safety of embolizing periarticular neovessels [23]. Critically, however, none of these models reproduces chronic immune-mediated synovitis: they represent degenerative, mechanical or chemically induced disease, and the histological consequences of embolization in a joint driven by adaptive immunity remain uncharacterised.

This differs mechanistically from radiofrequency ablation or cryoneurolysis, which act on sensory nerves rather than the vascular component of the synovial microenvironment [24, 25], and from classical embolization for haemorrhage or tumours, which seeks occlusion rather than pruning [26–28].

Biological plausibility, however, is not a clinical indication, and the question of whether vascular modulation in a rheumatoid joint translates into meaningful and durable benefit, without disrupting systemic disease control or introducing ischaemic risk, cannot be answered by mechanistic reasoning alone. The procedural literature offers the closest available proxy, although it is dominated by knee OA [29]. Open-label cohorts report consistent analgesic signals, i.e. pooled pain reductions of ≈30 points on a 0–100 scale at 6–12 months and 78–92% of patients achieving a clinically meaningful response (≥50% pain reduction) by 12 months [30]. Sham-controlled trials tell a more cautious story: two of the three published studies failed to demonstrate the superiority of TAE over the sham procedure at medium-term follow-up [31–33]. This is not a failure of concept; it is a signal that placebo response is large, patient selection matters enormously and procedural research in musculoskeletal disease has not yet reached methodological maturity.

For rheumatology, the OA experience is most instructive not for its efficacy estimates but for what it offers on two fronts: methodological lessons and a benchmark of procedural safety. Methodologically, it teaches the need to anchor patient selection to objectively documented hypervascularity, to extend outcomes beyond pain reduction and to build sham controls where feasible [34–36]. In terms of safety, the profile in degenerative musculoskeletal conditions has become increasingly characterized. Reported adverse events are predominantly minor and self-limited, including transient skin discoloration, post-procedural pain and access site haematoma, whereas serious complications such as skin necrosis or clinically significant non-target embolization have been reported only rarely [37, 38]. The choice of embolic agent is a further unresolved variable, and one that matters chiefly for safety rather than efficacy. A systematic review and meta-analysis dedicated specifically to this comparison in OA found that temporary (imipenem/cilastatin) and permanent microsphere agents produced statistically equivalent pain outcomes but their safety profiles were not equivalent: focal asymptomatic osteonecrosis was reported exclusively in a permanent microsphere series (8% of treated knees) and in no imipenem/cilastatin series and cutaneous complications were more frequent with permanent particles [39]. Part of this difference appears technique- and era-dependent—contemporary calibrated microspheres (<100 µm) used with cone-beam CT guidance achieve skin complication rates comparable to imipenem/cilastatin—rather than strictly agent intrinsic. The mechanistic concern nonetheless remains that permanent agents, by producing durable rather than transient devascularisation, could carry a greater risk of persistent ischaemic injury or impaired tissue recovery.

In the absence of any disease-specific comparison, embolic agent selection—together with prospective imaging surveillance for osteonecrosis when permanent agents are used—should be treated as a central safety question in future study designs rather than a settled technical detail. Whether these signals translate to inflammatory arthritis is an open question, and the differences are not trivial. Patients with immune-mediated disease differ from typical OA cohorts in ways directly relevant to procedural safety: many receive systemic immunosuppression, which might alter healing and infection risk [40]; the microvascular biology of chronic synovitis differs from that of a degenerative joint; and the interaction between embolization-induced ischaemia and ongoing immune-mediated inflammation is uncharacterized. These are not reasons to consider TAE unsafe in inflammatory arthritis, they are reasons why its safety cannot be inferred from the OA literature.

## Where the question arises and where it might lead

Some clinical scenarios make the question of TAE in inflammatory arthritis biologically coherent and clinically proportionate, others do not. The contexts outlined below (Table 1) span a spectrum, from situations encountered in everyday rheumatology practice, where the question of fit is most pressing, to rarer settings where the biological rationale for a vessel-targeted approach is exceptionally well defined. The aim is not to define indications. It is to mark out where rheumatology might begin engaging with a question that has, so far, surfaced mostly in case reports [1, 41–43].

**Table 1:** Clinical scenarios where questions about TAE in inflammatory arthritis are starting to surface.

Single-joint escape in otherwise controlled systemic disease
• One joint remains clinically or ultrasonographically active despite global remission or low disease activity—a dilemma of proportionality in which systemic escalation carries unchanged immunosuppressive risk for localised residual disease.
Constrained systemic escalation
• Persistent mono- or oligoarticular synovitis, tenosynovitis or enthesitis where systemic therapy is limited by active infection, malignancy, severe comorbidity or patient refusal, including severe needle phobia. The therapeutic gap is genuine; the procedural rationale is one of necessity rather than preference.
Immune checkpoint inhibitor–associated inflammatory arthritis
• A frontier where the need for localised steroid-sparing approaches is conceptually clear and procedural strategies are almost entirely untested. Multidisciplinary context is essential.
Haemophilic arthropathy
• Recurrent haemarthrosis driving synovial hypertrophy, pathological angiogenesis and a self-perpetuating cycle of microbleeding and inflammation. The hypervascular architecture of hypertrophic haemophilic synovium provides the most specific mechanistic target for vessel-directed embolization currently available in any inflammatory context.
Inflammatory arthritis with concomitant symptomatic OA
• Overlapping inflammatory and degenerative mechanisms complicate clinimetric interpretation and may bias escalation decisions toward the inflammatory component when the dominant pain driver is degenerative.
Chronic crystal-associated inflammatory arthritis
• Focal, treatment-refractory phenotypes in which evidence-based systemic options are limited and repeated intra-articular interventions are no longer appropriate or effective.

The scenario closest to mainstream rheumatology practice is single-joint escape in otherwise controlled systemic disease. A patient achieves remission or low disease activity on a bDMARD or tsDMARD, yet one joint remains clinically or ultrasonographically active. The conventional response is to increase or switch systemic therapy, exposing the patient to unchanged immunosuppressive risk in pursuit of localised benefit. The proportionality question is genuine. Where, if at all, might a vessel-targeted approach fit between an inadequate but tolerable status quo and a systemic switch whose incremental benefit is, by definition, marginal? This is the scenario where the question of fit matters most, and where the absence of comparative evidence is most consequential.

A related scenario is constrained systemic escalation: mono- or oligoarticular synovitis where adding or intensifying immunosuppression is limited by active infection, malignancy, major comorbidity or patient refusal. Here the therapeutic gap is genuine and poorly served by existing options. A vessel-targeted approach enters the discussion not because the evidence supports it but because the clinical problem demands a vocabulary for it, and because the absence of such a vocabulary risks ad hoc adoption driven by clinical necessity ahead of a coherent methodological framework.

A speculative but clinically grounded extension of this scenario is enthesitis, the core lesion of PsA and SpA, which may remain locally refractory at a single site despite otherwise acceptable global control. Whether a vessel-targeted approach is transferable from synovitis-dominant disease to enthesitis is a genuine question, and preliminary procedural experience suggests it is at least technically feasible: TAE has been applied to refractory tendinopathies and enthesopathies with sustained pain relief and no major adverse events [44], and we have observed embolization of the hypervascularised enthesis in refractory pes anserinus enthesitis, with a reduction of soft tissue signal and near-complete resolution of perientheseal bone marrow oedema on follow-up MRI [45]. Related tendinous targets, such as the gluteal enthesis in greater trochanteric pain syndrome, have likewise been embolised safely [46]. These observations, however, derive from degenerative, mechanical or overuse enthesopathy, not from the immune-mediated enthesitis of SpA, in which the dominant drivers are innate immune activation along the IL-23/IL-17 axis and biomechanical stress at the insertion [47]. Whether attenuating entheseal hypervascularity meaningfully modifies a lesion sustained by those mechanisms is entirely untested, and the anatomy of many entheses—together with the relatively hypovascular nature of normal tendon—may raise different safety considerations from periarticular synovial embolization. We therefore raise enthesitis not as a candidate indication but as a delimited, and biologically instructive, research question.

A distinct and particularly challenging clinical setting is immune checkpoint inhibitor–associated arthritis, the most common musculoskeletal immune-related adverse event of checkpoint inhibitor therapy. Affecting ≈4% of treated patients, immune checkpoint inhibitor–associated arthritis predominantly presents as polyarthritis, but oligo- and monoarticular phenotypes occur and may persist long after immunotherapy discontinuation [48]. The therapeutic dilemma in this setting is unusually acute. Systemic corticosteroids, the conventional first line, may compromise antitumour efficacy at daily doses >10 mg of prednisone equivalent, while bDMARDs and tsDMARDs, although effective for arthritis control, have been associated with a shorter time to cancer progression in real-world cohorts [49]. These constraints generate a genuine therapeutic vacuum in which localised, immunologically minimal strategies are not only attractive but conceptually warranted. Adding biological plausibility, elevated VEGF levels have been described in immune checkpoint inhibitor–associated arthritis serum and synovium and correlate with disease severity—a signal that points to vascular pathways as potentially relevant in this setting [48].

This vascular thread becomes most explicit in haemophilic arthropathy. Although a less frequent encounter in rheumatology practice, it presents arguably the strongest biological rationale for vessel-targeted intervention in any inflammatory joint context. The most biologically grounded scenario is persistent single-joint disease where the dominant feature is a self-sustaining local vascular niche, and haemophilic arthropathy illustrates the extreme version: hypertrophic, hypervascular synovium harbouring fragile neovessels prone to recurrent microbleeding, creating a cycle of proliferation, inflammatory mediator release and further angiogenesis that can persist independent of the underlying coagulopathy [50, 51]. The therapeutic logic of vessel-targeted embolization here is direct: reduce the pathological vascular network and potentially interrupt the cycle, a rationale supported by encouraging findings from small case series [52]. Whether analogous self-sustaining niches develop in a subset of patients with RA or PsA presenting as single-joint escape is not an established fact but can be a testable hypothesis.

Two further contexts sit at the margin of this discussion, sharing a structural feature that is conceptually interesting: in both, inflammation is real but its driver is mixed or atypical, blurring the rationale for conventional escalation. The first is inflammatory arthritis with concomitant symptomatic OA, in which degenerative and inflammatory mechanisms overlap and may bias clinical scores toward apparent active disease, leading to escalation decisions that target the inflammatory component when the dominant pain driver is degenerative. The second is chronic crystal-associated arthritis that has evolved into a focal, refractory phenotype with limited evidence-based systemic options. Neither case fits the standard treat-to-target template, and in both the question of where a localised intervention might fit—if anywhere—is worth posing.

What unites these scenarios, in terms of practical reasoning, is not just shared biology but a shared positioning challenge. The question is not whether TAE could ever replace DMARDs—biologically implausible and clinically inappropriate—nor whether it could supplant intra-articular glucocorticoids, whose rapid action and low cost remain unmatched at the bedside. The relevant comparators are the next-step interventions: switching bDMARDs or tsDMARDs in patients already on adequate background therapy; surgical synovectomy in patients with a single refractory joint; or, for those who simply cannot escalate systemically, no further intervention at all.

Positioning TAE means specifying which of these comparators it might substitute for or complement, in which clinical phenotypes and under what evidentiary standards. Without that specification, even apparent successes remain isolated and the procedure risks finding its place inferred from where it is offered rather than designed from where it should be. The question of whether TAE might have a role is worth asking, provided it is approached within a framework of structured data collection and disciplined scepticism.

Positioning TAE also requires situating it against the other local procedures that already exist for refractory monoarthritis rather than against systemic escalation and surgery alone. The most established non-surgical option is radiosynoviorthesis, the intra-articular injection of a β-emitting radiocolloid (yttrium-90, rhenium-186 or erbium-169, according to joint size) that is phagocytosed by synovial lining macrophages, producing sclerosis and fibrosis of the inflamed synovium; it has been used for decades across RA, SpA and haemophilic arthropathy and is regarded as an alternative to surgical synovectomy in these settings [53]. Its conceptual target differs fundamentally from that of TAE: radiosynoviorthesis ablates the synovial tissue itself through local irradiation, whereas embolization modulates the vascular bed that sustains it. The practical requirements differ accordingly—radiosynoviorthesis depends on nuclear-medicine facilities, radiopharmaceutical licensing that varies between countries and post-injection joint immobilisation—as do the safety considerations, which centre on radiocolloid leakage, radiation exposure and a historically debated concern over chromosomal effects that has generally led to its avoidance in younger patients [53]. Surgical synovectomy, already the principal local alternative in this setting, targets the synovium by physical resection; it reliably relieves pain but is more invasive, carries a recovery and stiffness burden and is frequently followed by recurrence of synovitis and radiographic progression and so is not a substitute for systemic therapy [54]. Chemical synovectomy achieves ablation pharmacologically; each differs from TAE in target and risk profile. No comparative data position TAE against any of these, and we do not suggest superiority. The point is that a vessel-targeted approach would enter a field of pre-existing local options and its place, if any, must be defined relative to them, not in isolation.

## What any responsible investigation must build in

If these clinical questions are worth asking, they need infrastructure that case reports cannot provide. Because TAE would represent an occasional and highly selected intervention for inflammatory arthritis, systematic data capture should become the starting point rather than an afterthought. A registry-first approach with harmonised imaging endpoints, standardised definitions of indication and treatment failure and longitudinal follow-up would provide the minimum infrastructure needed to move beyond isolated experiences. Without this, each new case remains an anecdote. With it, patterns become visible, denominators become calculable and hypotheses become testable.

This infrastructure is inseparable from the question of who does what. In almost all settings, the procedure itself would be performed by interventional radiology, which owns the technical expertise in superselective catheterisation and embolic delivery. Rheumatology, by contrast, is best placed to define the indication, confirm that a single joint genuinely drives the clinical picture, exclude subclinical polyarticular disease and assess longitudinal outcomes against disease activity and imaging endpoints. Neither discipline can responsibly own the pathway alone: indication-setting without procedural expertise risks inappropriate case selection, while procedural capability without rheumatological governance risks precisely the uncritical diffusion this review cautions against. Any future use in inflammatory arthritis should therefore be structured as a formal cooperative model—shared written protocols, multidisciplinary case selection, joint definition of treatment failure and prospective registries maintained by both disciplines—rather than left to ad hoc referral. Defining that division of responsibility now, before practice patterns form, is itself part of the positioning effort this review argues for.

Patient selection must be anchored to objective evidence of hypervascular inflammation with power Doppler ultrasound or contrast-enhanced MRI, not clinical impression alone. Subclinical polyarticular disease must be actively excluded: imaging-detected synovitis at apparently quiescent sites can reclassify a clinical mono- or oligoarticular presentation and change the therapeutic calculus entirely [55, 56]. Outcomes must extend beyond pain to encompass imaging-based quantification of synovitis, joint-specific function and steroid-sparing potential. Given the magnitude of placebo response documented in OA trials, sham-controlled or delayed-intervention designs will eventually be essential to distinguish a genuine procedural effect from regression to the mean and natural disease fluctuation [57, 58]. Safety reporting must be prospective and explicit, particularly for ischaemic risk, vascular complications and the interaction between embolic agent selection and the immunological context of the treated joint.

The history of musculoskeletal procedural medicine offers cautionary precedents: vertebroplasty for vertebral compression fractures, arthroscopic debridement for knee OA and subacromial decompression for shoulder pain all diffused widely before sham-controlled trials revealed limited or absent benefit, and in each case revision of practice was slow and incomplete [59–61]. For a procedure as conceptually new to rheumatology as TAE, defining whether a legitimate role exists—and if so, where its positioning should lie—will necessarily be a gradual process. But this is precisely what allows the community to engage with the question on its own terms rather than inheriting the trajectory of other procedural fields, where diffusion preceded evidence and practice became entrenched before revision was possible.

## Conclusions

Monoarthritis is a clinical reality that rheumatology’s composite metrics dilute and its treatment frameworks leave poorly served [2]. One persistently inflamed joint can concentrate pain, functional disability and structural risk while a global score indicates target achievement. This review asks whether a vessel-targeted approach, currently at the outermost frontier of what our discipline has so far considered, deserves a deliberate positioning effort from our community rather than ad hoc adoption or reflexive dismissal.

Procedural innovation has a consistent track record of outpacing the evidence base when early case reports generate enthusiasm and practice shapes itself before data arrive. Mapping these questions now—which are biologically coherent, clinically proportionate and simply not yet answerable—is not a conservative reflex. It is the prerequisite for study designs that will eventually tell us whether TAE has any legitimate place in inflammatory arthritis, and if so, for whom.

The intention is not to proceduralize rheumatology. It is to ensure that when procedural medicine extends into our field, it does so on terms that rheumatology has had the chance to define.

## Data Availability

No new data were generated or analysed in support of this review. All data discussed derive from the published studies cited herein.
